# Endogenous and exogenous sex steroid hormones in asthma and allergy in females: A systematic review and meta-analysis

**DOI:** 10.1016/j.jaci.2017.11.034

**Published:** 2018-04

**Authors:** Nicola McCleary, Bright I. Nwaru, Ulugbek B. Nurmatov, Hilary Critchley, Aziz Sheikh

**Affiliations:** a Asthma UK Centre for Applied Research, Centre for Medical Informatics, Usher Institute of Population Health Sciences and Informatics, University of Edinburgh, Edinburgh, United Kingdom; b Clinical Epidemiology Program, Ottawa Hospital Research Institute, Ottawa, Ontario, Canada; c Krefting Research Centre, Institute of Medicine, University of Gothenburg, Gothenburg, Sweden; d Wallenberg Centre for Molecular and Translational Medicine, Institute of Medicine, University of Gothenburg, Gothenburg, Sweden; e Division of Population Medicine, School of Medicine, Cardiff University, Cardiff, United Kingdom; f Medical Research Council Centre for Reproductive Health, Queen's Medical Research Institute, University of Edinburgh, Edinburgh, United Kingdom

To the Editor:

Asthma and allergy are more common in males than in females during early childhood, but the incidence, severity, and impact on quality of life are greater in postpubertal females than in males.^1^, ^2^ Female sex steroid hormones may partly explain these differences.^1^, ^2^ In 2 previous systematic reviews, early menarche (<12 years) was associated with an increased asthma risk,^3^ whereas no significant association was found between menopause and asthma, although subgroup analyses indicated an increased risk in postmenopausal women using hormone replacement therapy (HRT).^4^ Consideration of other hormonal factors, along with the full spectrum of relevant outcomes, is necessary for a comprehensive appreciation of the underlying evidence base. We therefore undertook a systematic review investigating the role of endogenous and exogenous hormonal factors in the development and clinical expression of asthma and allergy in females.

Our methods were published a priori (PROSPERO: 2015:CRD42015026762).^5^ Further details are available in this article's Online Repository at www.jacionline.org. We included experimental and analytical epidemiological studies of females from puberty to adulthood (<75 years). Exposures were puberty, menarche, menstruation, menopause, hormonal contraceptives, and HRT. Primary outcomes were self-reported or objectively defined incidence or prevalence of asthma, asthma exacerbations, asthma hospitalizations, and asthma medication use.

We searched 11 bibliographic databases, databases of ongoing studies, and conference abstracts, and contacted experts for articles published between January 1990 and November 2015 with no language restrictions. N.M. and B.I.N. independently screened titles, abstracts, and full-text articles; extracted study data; and assessed risk of bias using the Cochrane Risk of Bias Tool (experimental studies) and the Effective Public Health Practice Project tool (observational studies). Discrepancies were resolved by discussion, or arbitration by A.S.

Adjusted effect estimates were combined in random-effects meta-analyses, performed using Stata release 14 (StataCorp, College Station, Tex). Meta-analyses were possible for studies on menarche, menstruation, menopause, hormonal contraceptives, and HRT. Stratified analyses were performed by body mass index and smoking for HRT studies.

Of 22,488 articles retrieved, 64 (reporting 57 studies; observational: 51; experimental: 6) were included with 554,293 participants analyzed (see references E5 and E10-E72 and Fig E1 in this article's Online Repository at www.jacionline.org). Study characteristics are available on request.

Detailed results are given in this article's Online Repository at www.jacionline.org; here, we present key findings. Compared with typical menarche (11-13 years), early menarche (<11 years) was associated with increased risk of new-onset (odds ratio [OR], 1.49; 95% CI, 1.14-1.94) and ever asthma (OR, 1.06; 95% CI, 1.03-1.10), whereas late menarche (>13 years) was associated with increased risk of ever (OR, 1.11; 95% CI, 1.07-1.15), but not new-onset asthma (OR, 1.13; 95% CI, 0.82-1.56) (Fig 1).

**Fig 1:**
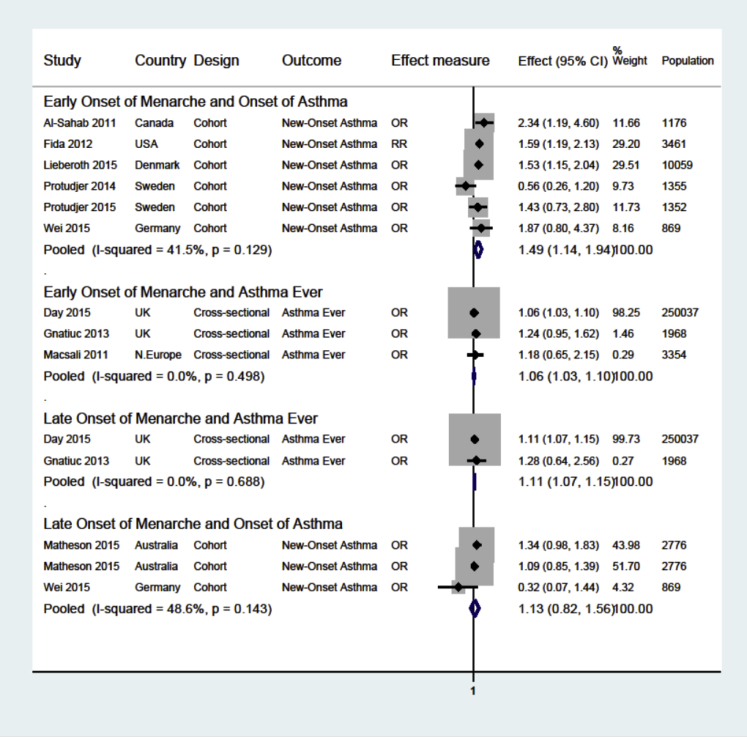
Meta-analyses of studies that investigated associations between onset of menarche and asthma and allergy in females. *N.Europe*, Northern Europe; *RR*, risk ratio. All effect estimates are adjusted. Weights are from random-effects analysis. Early menarche: <11 years; late menarche: >13 years; the comparator group in each analysis is typical menarche: 11 to 13 years.

Compared with regular menstruation, irregular menstruation was associated with increased risk of current asthma (past 12 months) (OR, 1.59; 95% CI, 1.23-2.05) (see Fig E2, *A*, in this article's Online Repository at www.jacionline.org), specifically for atopic (OR, 2.57; 95% CI, 1.66-3.98), but not nonatopic asthma (OR, 0.95; 95% CI, 0.54-1.65) (Fig E2, *B*).

Compared with premenopause, menopause onset was associated with increased risk of current asthma (OR, 1.25; 95% CI, 1.04-1.51) and current wheeze (OR, 1.16; 95% CI, 1.05-1.30), but not current allergic rhinitis (OR, 0.94; 95% CI, 0.81-1.10) (see Fig E3 in this article's Online Repository at www.jacionline.org).

Results for hormonal contraceptives were mixed, with both increased and decreased risks reported (Fig E3).

Compared with never use, ever HRT use (hazard ratio [HR], 1.37; 95% CI, 1.22-1.54), past use (HR, 1.41; 95% CI, 1.22-1.63), current use (HR, 1.48; 95% CI, 1.22-1.78), and current use of estrogen-only HRT (HR, 1.85; 95% CI, 1.50-2.28) were associated with increased risk of new-onset asthma (Fig 2). Current use was also associated with increased risk of current asthma (OR, 1.42; 95% CI, 1.18-1.70), and current wheeze (OR, 1.40; 95% CI, 1.22-1.61), but not current allergic rhinitis (OR, 1.27; 95% CI, 0.97-1.68) (Fig 2). The risk was higher in nonoverweight/nonobese and nonsmoking women than in overweight/obese and smoking women, respectively (see Fig E4 in this article's Online Repository at www.jacionline.org).

**Fig 2:**
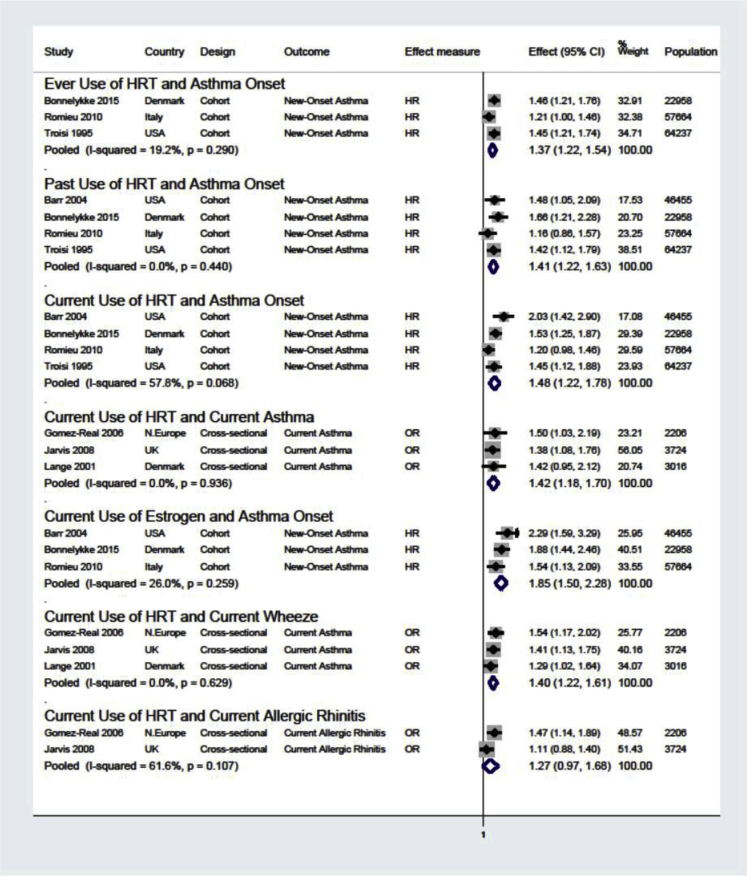
Meta-analyses of studies that investigated associations between the use of HRT and asthma and allergy in females. *N.Europe*, Northern Europe. All effect estimates are adjusted. Weights are from random-effects analysis. The comparator group in each analysis is never use.

Forty-one of the 51 observational studies had moderate risk of bias, whereas the rest had high risk; all 6 experimental studies had high risk of bias (data available on request).

This is the most comprehensive synthesis to date linking sex steroids to the development and expression of asthma and allergy in females. We followed recommended steps for undertaking a high-quality synthesis. However, the lack of high-quality experimental studies limited assessment of causality and relevance to patient care and policy. Although most epidemiological studies adjusted for key confounders, this was often not comprehensive. Many outcomes (eg, medication use, exacerbations, and hospitalizations) were not assessed, and so there is little evidence in relation to these.

Questions that remain to be addressed center on the influence of different types of sex steroids, dose and route of administration of exogenous sex steroids, and the underlying biologic mechanisms through which hormones may influence asthma and allergy. Early menarche and irregular menstruation are often signs of anovulation, indicating episodes of unopposed estrogen exposure to target organs and absences of progesterone exposure.^6^ Estrogen-only HRT was associated with asthma, whereas smoking, which may influence estrogen metabolism,^7^ had a protective effect. Although higher body mass index is generally associated with a more estrogenic state^8^ and also an increased risk of asthma,^9^ we found increased risk in both overweight/obese and nonoverweight/nonobese HRT users. At the cellular level, estrogen can have proinflammatory or anti-inflammatory effects, depending on cell type and location. Explanations for the various associations found are undoubtedly complex, and it is unlikely that all can be explained by one biological mechanism. Our results also suggest that atopy may be a contributing factor in some instances (irregular menstruation) but not others (menopause). The differences in findings for early and late menarche may be due to the different asthma outcomes investigated: alternatively, the effect observed in cross-sectional studies may reflect reverse causation. Further mechanistic work is required to elucidate any relationships, as are further longitudinal observational studies with detailed phenotyping of participants.

## Figures and Tables

**Fig E1:**
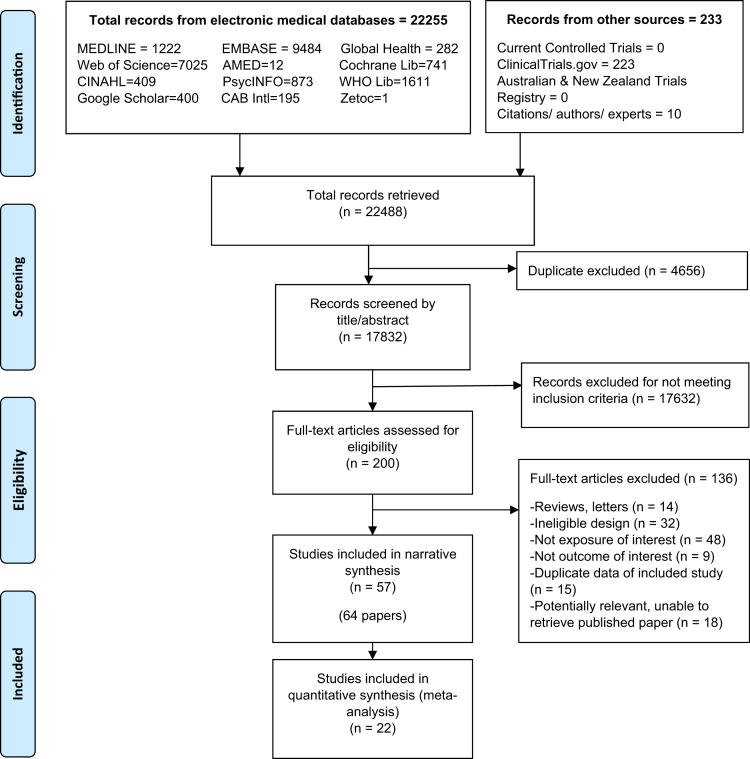
Preferred Reporting Items for Systematic reviews and Meta-Analyses (PRISMA) flow diagram summarizing the identification, screening, eligibility, and inclusion of studies that investigated associations between endogenous and exogenous sex steroid hormonal factors and asthma and allergy in females. For Google Scholar, the first 400 hits were selected for screening.

**Fig E2:**
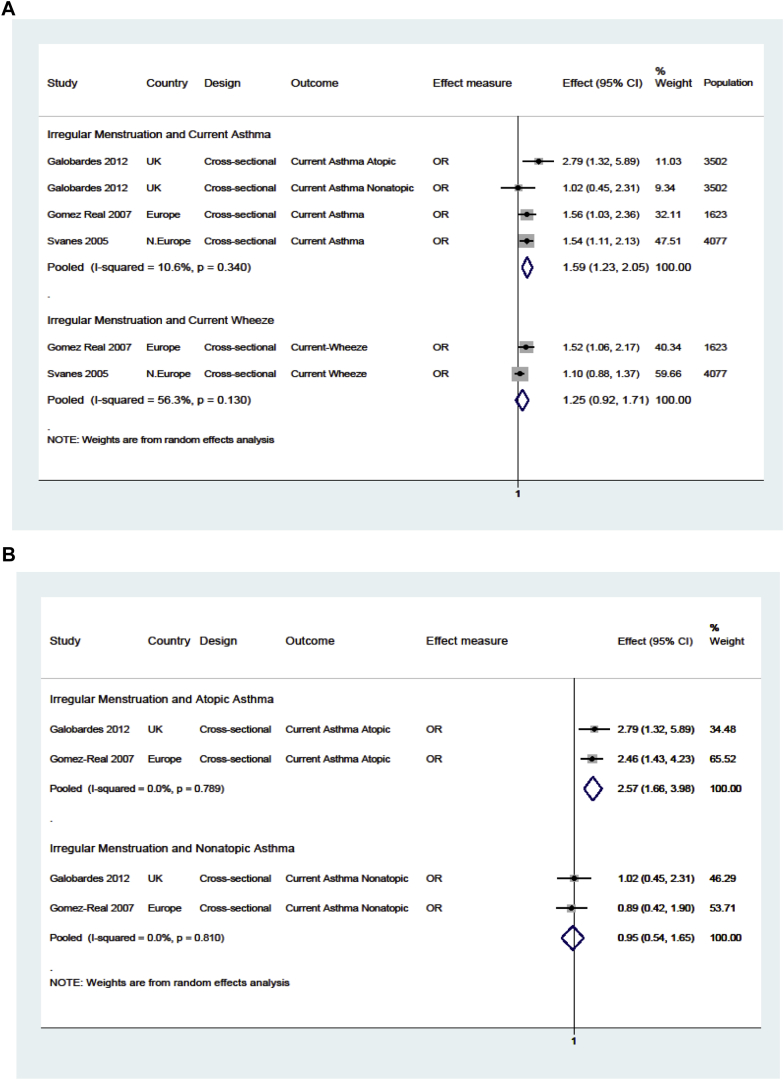
**A,** Meta-analyses of studies that investigated associations between irregular menstruation and asthma and allergy in females. **B,** Meta-analyses of studies that investigated associations between irregular menstruation and asthma in females, stratified by atopy. *N.Europe*, Northern Europe. All effect estimates are adjusted. The comparator group in each analysis is regular menstruation.

**Fig E3:**
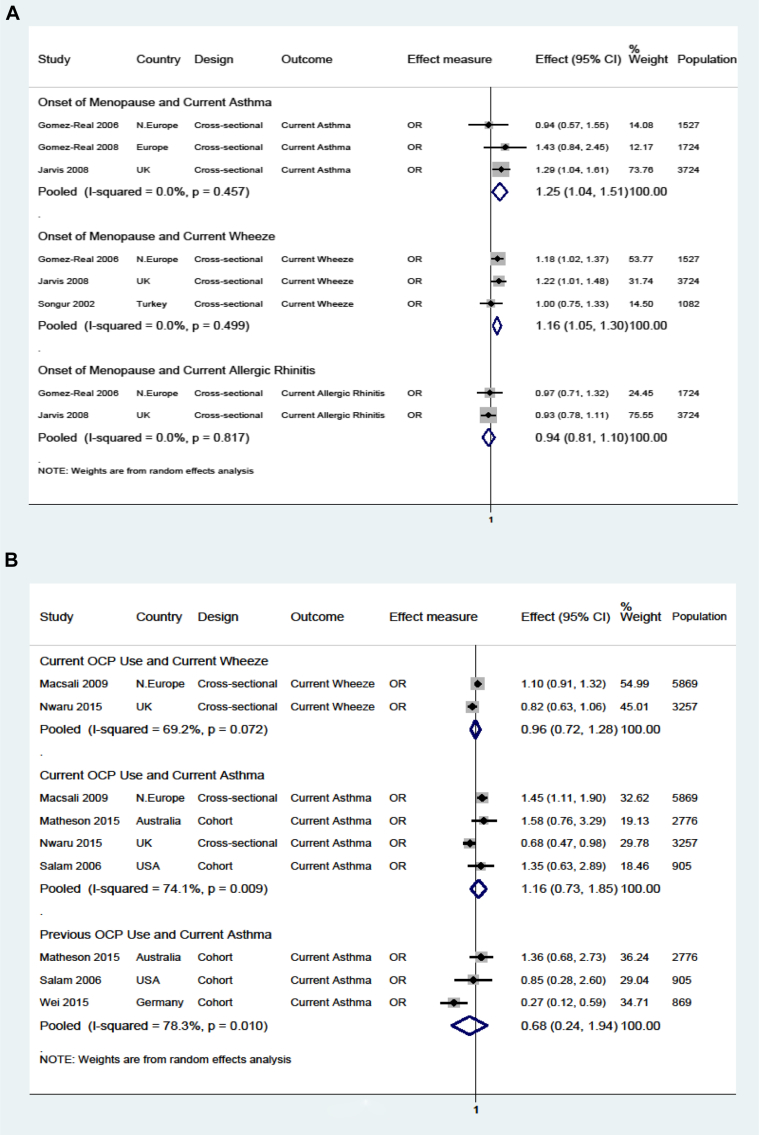
Meta-analyses of studies that investigated associations between onset of menopause and asthma and allergy in females **(A)** and between OCP use and asthma and allergy in females **(B)**. *N.Europe*, Northern Europe. All effect estimates are adjusted. The comparator group is premenopause **(A)**/never use **(B)**.

**Fig E4:**
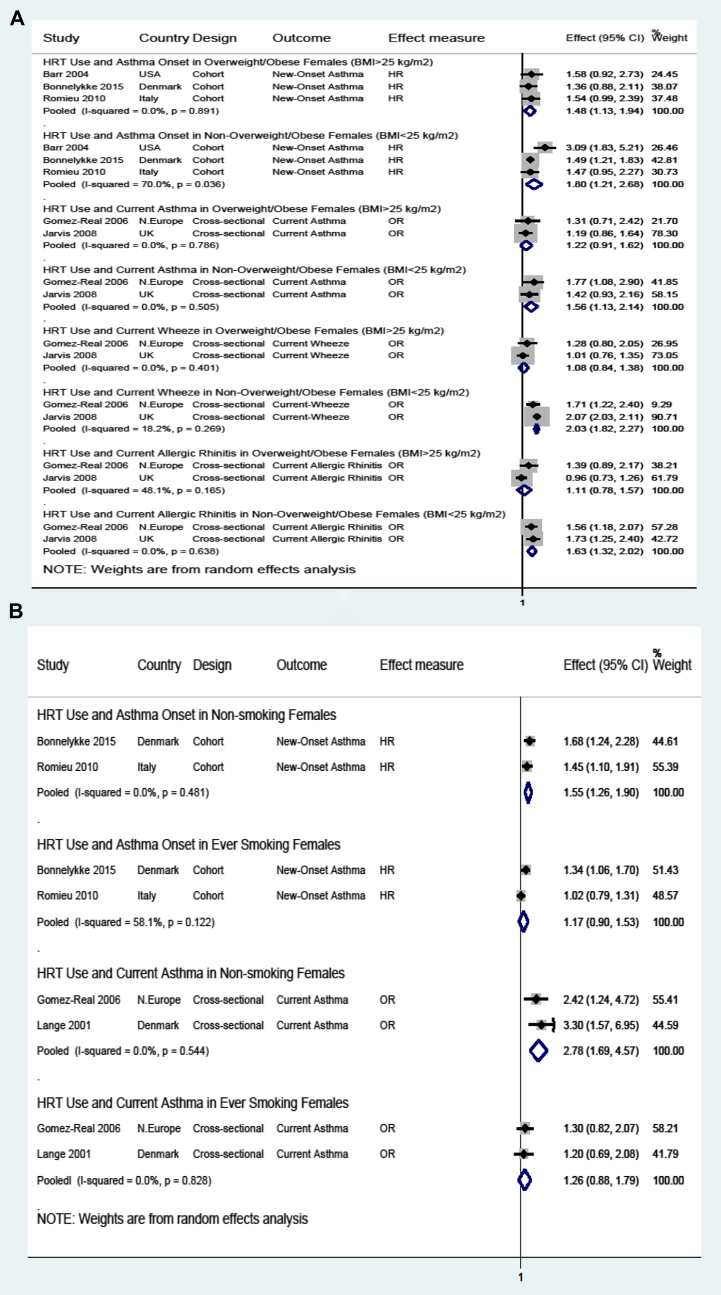
Meta-analyses of studies that investigated associations between the use of HRT and asthma and allergy in females, stratified by overweight/obesity **(A)** and smoking status **(B)**. *N.Europe*, Northern Europe. All effect estimates are adjusted. The comparator group in each analysis is never use.

## Methods

Our methods are detailed in full in PROSPERO (2016: CRD42015026762) and in our published protocol.^E1^

### Eligibility criteria

Experimental studies (randomized controlled trials [RCTs], quasi-RCTs, controlled-clinical trials [CCTs], controlled before-and-after studies, and interrupted time series designs) and analytical epidemiological studies (cohort, case-control, and cross-sectional studies) were eligible for inclusion. Females from puberty to adulthood (<75 years) were eligible. There was no fixed lower age limit of puberty; rather, we used the definitions given in each article. Exposures were endogenous (ie, puberty, menarche, menstruation, menopause) and exogenous (ie, hormonal contraceptives and HRT) hormonal factors. Categories of exposures were defined according to the definitions used in included studies (eg, early vs typical menarche, irregular vs regular menstruation, pre- vs postmenopause, use vs nonuse of hormonal contraceptives, and duration of use of HRT). Our primary outcomes were self-reported or objectively defined measures of incidence or prevalence of asthma, asthma exacerbations, asthma hospitalizations, and use of asthma medications: again, categories were defined according to the included studies (eg, has asthma diagnosis or not, ever hospitalized for asthma). Our secondary outcomes were wheeze, atopic dermatitis/eczema, allergic rhinitis, urticaria, angioedema, food allergy, anaphylaxis, atopic sensitization, indicators of lung function, and asthma-specific quality of life.

### Search strategy and study selection

We searched 11 bibliographic databases (MEDLINE, EMBASE, Cochrane Library, ISI Web of Science, CINAHL, Google Scholar, AMED, Global Health, PsycINFO, CAB International, and WHO Global Health Library) for articles published between January 1990 (because relevant studies were published from the mid-1990s) and November 2015 with no language restrictions. An example search strategy is provided in Appendix E1. References cited in included studies were screened, and international experts in the field were contacted. Unpublished and ongoing studies were searched using ISI Conference Proceedings Citation Index via Web of Knowledge, ZETOC (British Library), Current Controlled Trials, ClinicalTrials.gov, and the Australian and New Zealand Clinical Trials Registry. Titles and abstracts of retrieved articles and full-text copies of potentially relevant studies were screened independently by 2 reviewers (N.M. and B.I.N.). Discrepancies were resolved by discussion, or arbitration by a third reviewer (A.S.). A record of reasons for rejection at the full-text screening stage was kept and interreviewer agreement was assessed using the Kappa statistic.^E2^

### Data extraction and risk-of-bias assessment

A data extraction form was developed, independently piloted by N.M. and B.I.N., and refined before use. N.M. and B.I.N. independently extracted study data and completed risk-of- bias assessments. Discrepancies were resolved by discussion, or arbitration by A.S. The risk of bias in experimental studies was assessed using the Cochrane Risk of Bias Tool.^E3^ For observational studies, the Effective Public Health Practice Project tool was used (www.ephpp.ca/tools.html). This tool was adapted for use, informed by the Research Triangle Institute item bank.^E4^ Data extraction and risk-of-bias assessment were undertaken by N.M. and U.N. for the study authored by B.I.N. and A.S.^E5^

### Data analysis and reporting

Descriptive tables were produced to summarize the literature and characteristics of studies contributing to the evidence. Effect estimates from studies judged to be reasonably homogeneous in terms of their clinical, methodological, and statistical aspects were combined in random-effects meta-analyses using the inverse variance method.^E6^ Meta-analysis was possible only with observational epidemiological studies and not with experimental studies, which were of low quality and heterogeneous. The studies on puberty were heterogeneous; hence, no meta-analysis was done; instead, we undertook a narrative synthesis of these studies. Meta-analyses were performed for studies on menarche, menstruation, menopause, hormonal contraceptives, and HRT. Studies eligible for meta-analyses reported 1 of the following effect measures: HR, risk ratio, or OR. We included only adjusted estimates in the meta-analyses. For studies of HRT, stratified analyses were performed for body mass index (non vs overweight/obese) and smoking (non vs ever-smoker). Heterogeneity was quantified using the *I*^2^ statistic. To enhance comparability between studies that categorized any of the exposures as binary, estimates from exposure categories in studies that used multiple exposure categories were collapsed using the Mantel-Haenszel approach^E7^ before combining in meta-analyses. Evidence of publication bias was assessed using Funnel plots and the Egger test.^E8^ All tests were 2-sided, and *P* < .05 was considered statistically significant. Analyses were performed using Stata release 14 (StataCorp). Reporting followed the recommendations of the Preferred Reporting Items for Systematic reviews and Meta-Analyses (PRISMA) checklist.^E9^

## Results

### Study selection

A total of 22,488 records were retrieved, of which 64 articles (reporting 57 studies) met the criteria to be included in the review.E5, E10, E11, E12, E13, E14, E15, E16, E17, E18, E19, E20, E21, E22, E23, E24, E25, E26, E27, E28, E29, E30, E31, E32, E33, E34, E35, E36, E37, E38, E39, E40, E41, E42, E43, E44, E45, E46, E47, E48, E49, E50, E51, E52, E53, E54, E55, E56, E57, E58, E59, E60, E61, E62, E63, E64, E65, E66, E67, E68, E69, E70, E71, E72 In total, 554,293 participants were analyzed across the included studies. Of the 57 studies, 22 were included in at least 1 meta-analysis. The 2 reviewers agreed on 99% of the records at the title and abstract screening stage (*κ* = 0.62 indicating good or substantial agreement) and 88% at the full-text article screening stage (*κ* = 0.76 indicating good or substantial agreement).^E2^ Articles in non-English languages (ie, French,^E67^ Turkish,E10, E54 and RussianE11, E12) were translated. The literature search and screening process are summarized in Fig E1.

### Characteristics of included studies

Twenty-six studies exclusively investigated endogenous hormones, 20 exclusively investigated exogenous hormones, and 11 investigated both. Of the 37 studies on endogenous hormones, 9 focused on puberty,E13, E24, E28, E33, E35, E53, E54, E56, E67, E70 22 on menarche,E14, E21, E23, E24, E25, E29, E30, E31, E34, E37, E38, E43, E44, E45, E48, E50, E53, E54, E56, E57, E60, E62, E63, E64, E71, E72 5 on menstruation,E22, E25, E30, E43, E44, E45, E66 and 11 on menopause.E15, E17, E27, E32, E36, E39, E40, E42, E43, E44, E45, E62, E63, E64, E68, E69 Of the 31 studies on exogenous hormones, 17 focussed on hormonal contraceptives (15 observationalE23, E26, E29, E32, E37, E42, E47, E49, E5, E50, E52, E60, E61, E62, E63, E64, E69, E71 and 2 experimental studiesE46, E58) and 18 on HRT (14 observationalE11, E12, E16, E18, E19, E32, E36, E39, E40, E41, E42, E47, E51, E59, E63, E69 and 4 experimental studiesE10, E20, E55, E65). Asthma diagnosis was a key outcome across studies, principally assessed on the basis of self-reports or parent reports. Our other primary outcomes were rarely assessed: 6 studies assessed asthma exacerbations, asthma hospitalizations, or use of asthma medications. Secondary outcomes included self-reported atopic dermatitis/eczema, wheeze, allergic rhinitis, and food allergy; measures of lung function using spirometry; and specific/total IgE assessed using serum samples. Detailed characteristics of included studies are available on request.

### Associations between endogenous sex hormones and risk of asthma and allergy

Across studies, early onset of puberty was associated with an increased risk of asthma, whereas late onset of puberty appeared to be protective (data available on request). Age at menarche was grouped around the age of 11 to 13 years across most studies, with early menarche typically defined as below this age and late menarche as above. Compared with typical menarche (11-13 years), early menarche was associated with increased risk of new- onset asthma (OR, 1.49; 95% CI, 1.14-1.94) and ever asthma (OR, 1.06; 95% CI, 1.03-1.10) (Fig 1), whereas late menarche was associated with ever asthma (OR, 1.11; 95% CI, 1.07-1.15), but not new-onset asthma (OR, 1.13; 95% CI, 0.82-1.56) (Fig 1).

Most menstruation studies focused on the impact of irregular menstruation. Limited information regarding the definition of irregular menstruation was provided in the studies included in meta-analyses, with regularity of menstruation categorized on the basis of self-reports of whether or not menstruation was regular, or whether cycle length was greater than 32 days.E30, E44, E66 Compared with regular menstruation, irregular menstruation was associated with increased risk of current asthma (having had asthma in the past 12 months) (OR, 1.59; 95% CI, 1.23-2.05), but not current wheeze (OR, 1.25; 95% CI, 0.92-1.71) (Fig E2, *A*). Stratifying by atopic status, irregular menstruation was associated with current atopic asthma (OR, 2.57; 95% CI, 1.66-3.98), but not with current nonatopic asthma (OR, 0.95; 95% CI, 0.54-1.65) (Fig E2, *B*).

Compared with the premenopausal period, onset of menopause was associated with increased risk of current asthma (OR, 1.25; 95% CI, 1.04-1.51) (Fig E3), and with an increased risk of new-onset asthma in one study,^E68^ but a decreased risk in another.^E69^ Each type of menopause (natural vs surgical) was associated with an increased risk of current asthma in one study,^E36^ but a decreased risk of new-onset asthma in another study.^E69^ One study found a significantly higher exacerbation rate in women who developed asthma around the time of their menopause than in women with preexisting asthma.^E15^ Another found that exacerbations were reported most frequently by women in early postmenopause, followed by those in late postmenopause or the menopausal transition, and those who were premenopausal; however, no significance tests were performed.^E68^ This study found the same pattern of reporting for current asthma medication use.^E68^ Onset of menopause was associated with increased risk of current wheeze (OR, 1.16; 95% CI, 1.05-1.30), but not current allergic rhinitis (OR, 0.94; 95% CI, 0.81-1.10) (Fig E3).

### Associations between exogenous sex hormones and risk of asthma and allergy

Results of studies on use of hormonal contraceptives were mixed, with both increased and decreased risks reported across studies (data available on request). Compared with never use, neither current (OR, 1.16; 95% CI, 0.73-1.85) nor past combined oral contraceptive pill (OCP) use (OR, 0.68; 95% CI, 0.24-1.94) was associated with current asthma (Fig E3). Although previous use of oral contraceptives was associated with an increased risk of new-onset asthma in one study,^E69^ a decreased risk after use of hormonal contraceptives was reported in another (hormonal compositions were not specified).^E71^ In one study, use of any hormonal contraceptive was associated with increased risk of having 3 or more asthma or wheeze care episodes in the past 12 months.^E5^ In another, women using the combined OCP were less likely to report using inhaled corticosteroid medication than women not using OCP.^E61^ One study investigated the role of types of hormonal contraceptives (combined OCP and progesterone-only preparations compared with nonuse), but found no association with the risk of either current asthma or current asthma or wheeze care episodes.^E5^ The duration of use of hormonal contraceptives was generally not associated with any outcome (data available on request). Current combined OCP use was not associated with current wheeze (OR, 0.96; 95% CI, 0.72-1.28) (Fig E3). Two experimental studies (CCTs) compared OCP to no OCP: mean FEV_1_/forced vital capacity % was significantly greater in the intervention group than in the control group in one study^E46^; the opposite occurred in the other.^E58^

Compared with never use, ever use of any HRT (HR, 1.37; 95% CI, 1.22-1.54), past use of any HRT (HR, 1.41; 95% CI, 1.22-1.63), current use of any HRT (HR, 1.48; 95% CI, 1.22-1.78), and current use of estrogen-only HRT (HR, 1.85; 95% CI, 1.50-2.28) were associated with increased risk of new-onset asthma (Fig 2). Current use was also associated with increased risk of current asthma (OR, 1.42; 95% CI, 1.18-1.70) (Fig 2). One study found an increased risk of first-ever asthma hospitalization with ever, previous, and current use of HRT (but not when HRT had been tried for <6 months); use of estrogen-only, sequential, and continuous HRT; as well as increased risk for every 5 years of HRT use.^E18^ Another found a lower exacerbation rate in women using combined HRT than in those not using HRT.E11, E12 Two studies found no association between asthma medication use and HRT use.E39, E47 Current use of HRT was associated with increased risk of current wheeze (OR, 1.40; 95% CI, 1.22-1.61), but not current allergic rhinitis (OR, 1.27; 95% CI, 0.97-1.68) (Fig 2). Stratified analyses indicated increased risk of asthma onset in both nonoverweight/nonobese and overweight/obese women using HRT; however, HRT use was associated with current asthma, current wheeze, and current allergic rhinitis in nonoverweight/nonobese but not overweight/obese women (Fig E4). Stratified analyses also indicated increased risk of asthma onset and current asthma in nonsmoking, but not ever-smoking women taking HRT (Fig E4). In the 4 experimental studies (1 CCT and 3 RCTs) that investigated the effects of different HRT regimens (comparing combined and estrogen-only regimens to placebo or no HRT) on lung performance, results were mixed.E10, E20, E55, E65

### Risk of bias within studies

Overall, 41 of 51 observational studies were graded moderate risk of bias, whereas the rest were graded high risk of bias. All 6 experimental studies had high risk of bias. Overall and domain-specific risk-of-bias ratings for each study are available on request. Funnel plots indicated more symmetry for studies on menstruation and HRT than for studies on menarche, menopause, and hormonal contraceptives (available on request). The associated *P* values for Egger test were as follows: menstruation *P* = .295; HRT *P* = .999; menarche *P* = .108; menopause *P* = .831; hormonal contraceptives *P* = .057.

## Appendix E1 MEDLINE search strategy—November 11, 2015

1 exp Puberty/or puberty.mp.
2 exp Menarche/or menarche.mp.
3 exp Menstruation/or exp Menstruation Disturbances/or menstruation.mp.
4 exp Menopause, Premature/or exp Postmenopause/or menopause.mp.
5 sex hormones.mp. or exp Gonadal Steroid Hormones/or exp Estrogens/or estrogens.mp. or exp Progesterone/or progesterone.mp. or Testosterone Congeners/or testosterone.mp. or exp Testosterone/or Testosterone Propionate/
6 exp Contraceptive Agents/or contraceptives.mp. or exp Contraceptives, Oral/or oral contraceptives.mp. or exp Contraceptives, Oral, Combined/or combined oral contraceptives.mp. or exp Medroxyprogesterone Acetate/or exp Contraceptive Agents, Female/or exp Contraceptives, Oral, Hormonal/or exp Contraception/or hormonal contraceptives.mp. or exp Ethinyl Estradiol/
7 exp Hormone Replacement Therapy/or exp Estradiol/or hormone replacement therapy.mp.
8 1 or 2 or 3 or 4 or 5 or 6 or 7
9 exp Asthma/or asthma.mp.
10 wheeze.mp.
11 exp Dermatitis, Atopic/or atopic eczema.mp.
12 exp Hypersensitivity, Immediate/or exp Hypersensitivity/or atopy.mp. or allergy.mp. or atopic sensitisation.mp. or allergic sensitisation.mp.
13 exp Rhinitis, Allergic, Seasonal/or exp Rhinitis, Allergic, Perennial/or exp Allergens/or allergic rhinitis.mp.
14 exp Conjunctivitis, Allergic/or Rhinoconjunctivitis.mp.
15 exp Conjunctivitis, Allergic/or Rhinoconjunctivitis.mp.
16 exp Urticaria/or urticarial.mp.
17 exp Angioedema/or angioedema.mp.
18 exp Food Hypersensitivity/or food allergy.mp.
19 exp Anaphylaxis/or anaphylaxis.mp.
20 lung function.mp.
21 airway function.mp. or exp Bronchial Hyperreactivity/
22 exp Forced Expiratory Volume/or forced expiratory volume in 1 second.mp.
23 exp Peak Expiratory Flow Rate/or peak expiratory flow.mp.
24 9 or 10 or 11 or 12 or 13 or 14 or 15 or 16 or 17 or 18 or 19 or 20 or 21 or 22 or 23
25 8 and 24
26 limit 25 to female
